# Impact of concordance between basal LH/FSH ratio and testosterone levels on pregnancy outcomes in patients with PCOS undergoing IVF-ET cycle: a retrospective cohort study

**DOI:** 10.1007/s40618-026-02902-7

**Published:** 2026-06-13

**Authors:** Hongyi Xu, Jingyou Li, Si Qing Yin, Qi Chen, Jiarong Tian, Xin Chen, Xin Zhang, Xin Li, Honglu Diao, Ying Zhang

**Affiliations:** 1 Reproductive Medicine Center, Renmin Hospital, Hubei University of Medicine, Shiyan, China; 2 Hubei Clinical Research Center for Reproductive Medicine, Shiyan, People’s Republic of China; 3 Biomedical Engineering College, Hubei University of Medicine, Shiyan, People’s Republic of China; 4 Biomedical Research Institute, Hubei University of Medicine, Shiyan, People’s Republic of China; 5 Hubei Key Laboratory of Embryonic Stem Cell Research, Hubei University of Medicine, Shiyan, People’s Republic of China

**Keywords:** Polycystic ovary syndrome (PCOS), Pregnancy outcome, *In vitro* fertilization and embryo transfer (IVF-ET), LH/FSH, Testosterone (T)

## Abstract

**Objective:**

To investigate the association of testosterone levels on ovarian stimulation response in PCOS patients—stratified by serum LH/FSH ratio—undergoing IVF-ET.

**Methods:**

A total of 1,354 PCOS patients who underwent fresh embryo transfer were ultimately included in the study. we analyzed and classified it according to the basal LH/FSH ratio values into the following groups: the LH/FSH ≥ 2 group (412 cases) and the LH/FSH < 2 group (942 cases). According to the value of basal serum T level, there were 488 cases in the high T group (> 0.75 ng/ml) and 866 cases in the non-high T group (≤ 0.75 ng/ml), and the outcomes of embryo transfer cycles were compared among the patients in each group.

**Results:**

Based on the LH/FSH ratio and testosterone (T) levels, patients were divided into four groups: Group A (LH/FSH < 2, T ≤ 0.75 ng/ml), Group B (LH/FSH < 2, T > 0.75 ng/ml), Group C (LH/FSH ≥ 2, T ≤ 0.75 ng/ml), and Group D (LH/FSH ≥ 2, T > 0.75 ng/ml). There were no statistically significant differences in embryo quality among the four groups (*P* > 0.05). Regarding pregnancy outcomes, Group B had the lowest live birth rate (51.56%), which was significantly lower than those of Group A (61.87%), Group C (59.79%), and Group D (68.10%)(*P* ≤ 0.05). Meanwhile, Group B had the highest miscarriage rate (22.38%), significantly higher than the other three groups (10.98%, 12.43%, and 12.90%) (*P* ≤ 0.05). After adjusting for confounding factors, logistic regression analysis using Group A as the reference showed that Group B had a significantly increased risk of miscarriage (*OR* = 2.200, 95% *CI* = 1.325–3.653, *P* = 0.002) and a significantly lower live birth rate (*OR* = 0.719, 95% *CI* = 0.520–0.994, *P* = 0.046).

**Conclusion:**

In patients with PCOS, a high LH/FSH ratio accompanied by elevated testosterone levels was not strongly associated with the likelihood of achieving a live birth. However, in the subgroup with an LH/FSH ratio ≤ 2, elevated testosterone levels may be associated with a reduced likelihood of live birth and an increased risk of miscarriage.

## Introduction

Polycystic ovary syndrome (PCOS) is primarily defined by ovulatory dysfunction, clinical or biochemical hyperandrogenism, and polycystic ovarian morphology [1, 2]. The syndrome exhibits considerable heterogeneity, and its underlying pathogenesis remains incompletely understood [3, 4]. A core feature of PCOS is endocrine dysfunction [5, 6], often manifesting as altered gonadotropin secretion. This is driven by factors such as excessive hypothalamic gonadotropin-releasing hormone (GnRH) pulsatility and a disrupted ovarian estrogen feedback mechanism, leading to increased luteinizing hormone (LH) release alongside normal or reduced follicle-stimulating hormone (FSH) levels, consequently elevating the LH/FSH ratio [7, 8]. These aberrant gonadotropin levels further exacerbate hyperandrogenism (HA) [3, 9], thereby increasing testosterone (T) levels [10].

Studies have shown that elevated basal androgen levels are associated with reduced sustained pregnancy rates following in vitro fertilization (IVF) assisted reproduction, suggesting that androgens may be a predictive factor for miscarriage [11]. Basal T levels in non-PCOS patients do not reflect clinical pregnancy outcomes, while PCOS patients with HA have significantly lower cumulative live birth rates compared to the non-HA group [12]. Studies on the impact of LH/FSH ratio on pregnancy outcomes have not reached a consensus. Some reports indicate that LH levels and LH/FSH ratio in PCOS patients do not affect clinical outcomes in ovulation induction patients [13, 14]. Some studies suggest that higher baseline LH levels may affect pregnancy outcomes in PCOS patients [15], while others report that PCOS women with higher LH/FSH ratios have higher clinical pregnancy rates, live birth rates, and lower miscarriage rates [16–18].

In this study, we conducted a retrospective analysis of PCOS patients with inconsistent LH/FSH ratios and T levels, both at high and non-high levels. few previous studies have investigated the impact of different T levels corresponding to different LH/FSH ratios on pregnancy outcomes. Therefore, for patients with high LH/FSH ratios, we explored the effects of different T levels on pregnancy outcomes to provide evidence for clinical endocrine treatment in PCOS patients.

## Materials and methods

### Study subjects

The subjects of this study were patients undergoing ovulation induction between January 2015 and December 2023, aged 20 to 40 years, who had not received any estrogen-progestin therapy prior to receiving assisted reproduction. All participants received ovarian stimulation using the long protocol for pituitary down-regulation and were undergoing their first cycle of in vitro fertilization (IVF) or intracytoplasmic sperm injection (ICSI).The inclusion criteria were as follows: (1) use of the standard long protocol for ovarian stimulation at our center; (2) diagnosis of polycystic ovary syndrome (PCOS) according to the 2003 Rotterdam criteria; (3) age under 40 years; and (4) first-time IVF/ICSI treatment. Key exclusion criteria included: (1) cycles involving preimplantation genetic testing; (2) concurrent reproductive tract malformations, such as uterine anomalies or bicornuate uterus; (3) presence of reproductive tract infections or systemic diseases (e.g., diabetes, heart disease, or hypertension); (4) severe intrauterine adhesions; (5) oocyte cryopreservation cycles; (6) functional hypothalamic amenorrhea; and (7) other fresh cycles in which embryo transfer was not performed. The screening and inclusion process of the study participants is detailed in Flowchart 1. The study was approved by the Ethics Committee of the Affiliated People’s Hospital of Hubei Medical University (Approval No.: SYRMYY-2025-101).

**Fig. 1:**
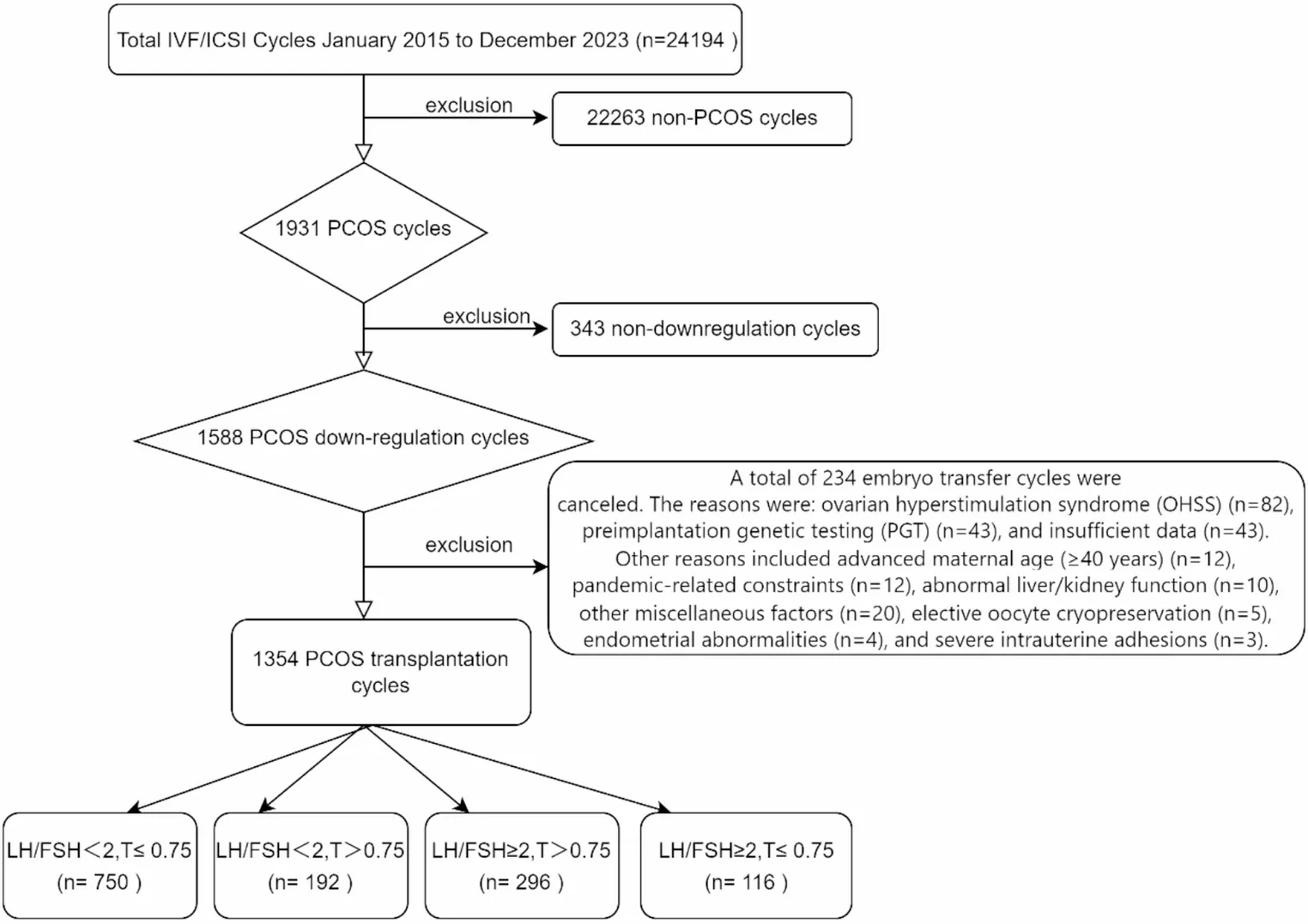
Data selection flowchart. IVF represents in vitro fertilization; ICSI represents intracytoplasmic sperm injection; PCOS represents polycystic ovary syndrome; PGT represents preimplantation genetic testing; T represents Testosterone; FSH represents serum follicle-stimulating hormone; LH represents luteinizing hormone; hCG represents human chorionic gonadotropin; OHSS represents ovarian hyperstimulation syndrome

Patients were stratified by their baseline LH/FSH ratio into a high-value (≥ 2) or non-high-value (< 2) group, and by baseline testosterone (T) levels into a high-T (> 0.75 ng/mL) or non-high-T (≤ 0.75 ng/mL) group. Based on these categorizations, patients with concordant parameters (both high or both low) were classified into the consistent group, whereas those with discordant parameters (one high and one low) were assigned to the inconsistent group (Fig. 1).

### Ovulation induction protocol

Down-regulated long protocol during the follicular phase: On day 3 of the menstrual cycle, perform an ultrasound (Diagnostic ultrasound machine, Aloka SSD-900, Japan) and measure serum anti-Mullerian hormone (AMH), follicle-stimulating hormone (FSH), luteinizing hormone (LH), estrogen (E2), progesterone (P), T, and prolactin (PRL). Administer 3.75 mg of long-acting triptorelin acetate (Decapeptyl; Ferring, SaintPrex, Germany) via intramuscular injection. 35–39 days later, perform an ultrasound and serum hormone tests. When E2 < 50 pg/ml, LH < 5 mIU/ml, FSH < 5 mIU/ml, *P* < 1 ng/ml, bilateral ovarian follicle diameter ≤ 5 mm, and endometrial thickness ≤ 5 mm, initiate exogenous recombinant gonadotropin (Gn) (rFSH, Gonal-f, Merck Serono, Switzerland) at 75–300 IU/day or human menopausal gonadotropin (HMG, Livzon Pharmaceutical, China). On day 4 of Gn administration, perform an ultrasound and serum hormone tests. Adjust the Gn dosage individually based on the test results. Subsequently, perform an ultrasound and serum FSH, LH, E2, and P tests every 2 days. When the follicle diameter reaches 16 mm, begin daily ultrasound and serum FSH, LH, E2, P tests. To trigger final oocyte maturation, either recombinant hCG (Merck Serono, Switzerland) at a dose of 250 µg or urinary hCG (Livzon Pharmaceutical, China) at a dose ranging from 1,000 to 2,000 IU was administered once two or more follicles had reached a pre-ovulatory size of 18–22 mm.

Luteal phase down-regulation protocol: Approximately one week before menstruation (equivalent to the mid-luteal phase), undergo an ultrasound examination to assess the condition of the uterus and ovaries, and measure serum LH, E2, and P levels. If E2 > 50 pg/ml and *P* > 5 ng/ml, indicating ovulation has occurred, begin subcutaneous injections of GnRHa at 0.05 mg/day or 0.1 mg/day. After 20 days of downregulation, perform an ultrasound examination and measure serum FSH, LH, E2, P, and β-HCG to determine if the downregulation criteria have been met (same as the long protocol for the follicular phase). If the criteria are met, adjust the GnRHa dose to 0.05 mg/day and concurrently administer Gn 75–300 IU/day. During the ovulation induction process, adjust the Gn dose based on follicle development monitoring results and serum hormone levels. When at least three dominant follicles reach a diameter of 20 mm, administer 250 µg of Aizawa or 2000 IU of HCG plus 250 µg of Aizawa, and retrieve oocytes 36–37 h later.

### Endocrine hormone determination

Collect 5 mL of blood from the elbow vein on the third day of menstruation (baseline FSH, LH, and T levels were measured on day 3 of the menstrual cycle before the start of the ovulation induction protocol)and on the day of hCG, centrifuge, and take the supernatant. Use a fully automated chemiluminescence method (Beckman Coulter UniCel DXi 800, USA) to measure FSH, luteinizing hormone (LH), estradiol (E2), and progesterone (P) levels, as well as T. The intra-assay and inter-assay variability for each reagent is within 10%. According to our center’s reference values, the normal range for testosterone is 0.1–0.75 ng/ml. When the T value exceeds 0.75 ng, it is considered high, as also reported in the literature [1, 19].

### In vitro fertilization, embryo transfer, luteal support

Following oocyte retrieval, the oocyte-coronacumulus complexes (OCCCs) are incubated for 2–4 h. Optimized sperm is then added to the culture droplet containing the OCCCs. After 4–5 h of co-incubation, the cumulus cells surrounding the oocyte are removed. For ICSI, the incubated OCCCs undergo hyaluronidase digestion followed by 1–2 h of incubation before ICSI injection. After 16–18 h, observation of the pronuclei confirms normal fertilization if both are in the 2PN stage. Patients undergo ultrasound-guided intrauterine transfer of 1–2 embryos on day 3 or 5 post-retrieval, with luteal support. Routine blood tests for E2 and P are performed on day 2 post-retrieval. Progesterone dosage and the addition of estradiol valerate or dydrogesterone are adjusted based on E2 and P levels. Blood hCG levels are measured 14 days post-transfer to confirm pregnancy. Values exceeding 5 IU/L indicate a biochemical pregnancy. A vaginal ultrasound at 4–5 weeks post-transfer showing fetal heart activity confirms clinical pregnancy.

### Observation indicators

Collect relevant medical history and physical examination data prior to ovarian stimulation, including both partners’ age, duration of infertility, height, weight, body mass index (BMI), AMH, duration of gonadotropin (Gn) use, and total Gn dosage. Embryo indicators: Usable embryo rate, high-quality embryo rate, blastocyst formation rate. Pregnancy Outcomes: hCG positive rate, clinical pregnancy rate, miscarriage rate, live birth rate. Relevant indicators are calculated as follows: Grade 3 embryo rate = Number of Grade 3 embryos on Day 3 / Number of normally fertilized oocytes × 100%; Usable embryo rate = Number of transferable embryos on Day 3 / Number of normally fertilized oocytes × 100%; Cystoid Embryo Formation Rate = Number of Cystoid Embryos Formed / Number of Cystoid Culture Embryos × 100%; Miscarriage Rate = Number of Miscarriage Cycles / Number of Clinical Pregnancy Cycles × 100%; Clinical Pregnancy Rate = Number of Clinical Pregnancy Cycles / Number of Fresh Transfer Cycles × 100%; Live Birth Rate = Number of Delivery Cycles with Live Births / Number of Embryo Transfer Cycles × 100%.

### Statistical methods

SPSS version 26.0 (IBM Corp., Armonk, NY, USA) was used for all statistical analyses. Normally distributed continuous variables are presented as mean ± standard deviation (x̄ ± s). Comparisons among multiple groups were performed using one-way analysis of variance (ANOVA). When ANOVA revealed significant differences, post-hoc pairwise comparisons were conducted with Bonferroni correction to adjust for multiple testing. Categorical variables are expressed as frequencies (percentages) and were compared using the chi-square test. Univariate analysis was first performed to identify potential confounding factors associated with clinical outcomes. Subsequently, multivariate logistic regression analysis was employed to examine the independent predictors of clinical outcomes after adjusting for these confounders; variables were selected based on clinical relevance and statistical significance in the univariate analyses. A two-tailed *P* value < 0.05 was considered statistically significant.

## Results

### Comparison of basic clinical characteristics among patient groups

A total of 1,354 patients undergoing ovulation induction for PCOS were included in this study. Among them, 942 cases had an LH/FSH ratio < 2, and 412 cases had an LH/FSH ratio ≥ 2. 866 cases had T ≤ 0.75 ng/ml, and 488 cases had T > 0.75 ng/ml. There were statistically significant differences between the four groups of patients in terms of female age, weight, AFC, bE2, and bLH (all *P* < 0.01). There were no statistically significant differences between the groups in terms of male age, duration of infertility, type of infertility, baseline AMH, ovulation induction protocol, bFSH, and bPRL levels (*P* > 0.05), as shown in Table 1.

**Table 1:** Comparison of basic information of patients before superovulation in each subgroup

Item	Synchronized		Unsynchronized		F/χ2 value	*P* value
	Group A	Group B	Group C	Group D		
No. of transplant cycles	750	192	296	116		
Female age (years)	28.99 ± 3.369	28.45 ± 3.147	28.40 ± 3.212	27.93 ± 3.059^abc^	7.118	0.000^***^
Male age (years)	30.81 ± 3.844	30.72 ± 4.105	30.56 ± 3.711	30.07 ± 3.656	1.964	0.117
Infertility type (%)					3.805	0.051
Primary	671	203	258	127		
Secondary	296	75	119	41		
BMI (kg/m^2^)	25.39 ± 4.32	26.06 ± 4.50	23.83 ± 4.10^ab^	24.79 ± 4.30^ab^	17.215	0.000^***^
Basal serum AMH level (µg/L)	9.93 ± 24.29	10.41 ± 5.14	11.68 ± 5.68	12.65 ± 5.92	1.476	0.219
Duration of infertility (year)	3.70 ± 2.42	3.65 ± 2.32	3.57 ± 2.18	3.54 ± 2.30	0.435	0.729
AFC	25.12 ± 8.53	27.19 ± 9.26	28.77 ± 8.78^a^	30.79 ± 9.98^abc^	29.380	0.000^***^
Basal hormone serum levels						
bFSH (IU/L)	6.92 ± 13.70	6.55 ± 1.82	5.99 ± 1.96	6.54 ± 1.72	0.771	0.510
bLH (IU/L)	7.24 ± 3.58	7.89 ± 3.50	17.12 ± 8.05^ab^	18.56 ± 7.97^ab^	465.248	0.000^***^
bPRL (ng/L)	13.78 ± 19.15	14.25 ± 24.07	15.73 ± 34.83	14.06 ± 20.07	0.580	0.628
bE2 (pg/ml)	44.85 ± 62.76	50.87 ± 38.71	68.10 ± 58.31^ab^	79.13 ± 147.90^ab^	17.169	0.000^***^

Notes: BMI represents body mass index; T represents testosterone; bFSH represents basal follicle-stimulating hormone; bLH represents basal luteinizing hormone; bE2 represents basal estradiol; AMH represents anti-Müllerian hormone

^a^*P* < 0.05, compared with group A; ^b^*P* < 0.05, compared with group B; ^C^*P* < 0.05, compared with group C

### ART treatment characteristics across four patient groups

There were no statistically significant differences between the four groups of patients in terms of ovulation induction protocols, endometrial thickness on the day of hCG injection, number of oocytes retrieved, and fertilization methods (all *P* > 0.05). There were statistically significant differences between the four groups in terms of Gn dosage and duration of treatment (*P* < 0.05), as shown in Table 2.

**Table 2:** Comparison of information on the process of superovulation in the cycle of assisted reproduction in each subgroup of patients

Item	Synchronized		Unsynchronized		F/χ2 value	*P* value
	Group A	Group B	Group C	Group D		
No. of transplant cycles	750	192	296	116		
No. receiving long protocol (%)					0.005	0.942
Follicular phase	839	237	293	138		
Luteal phase	128	41	84	30		
Dosage of Gn (IU)	2412.56 ± 1059.91	2377.42 ± 1010.90	1971.40 ± 848.70^ab^	2065.88 ± 931.18^ab^	21.102	0.000^***^
Duration of Gn (days)	12.05 ± 2.11	12.00 ± 2.32	11.54 ± 1.91^ab^	11.79 ± 2.29^ab^	5.524	0.001^***^
Endometrial thickness on the day of Hcg injection(mm)	11.58 ± 2.33	11.40 ± 2.26	11.48 ± 2.39	11.25 ± 2.27	0.781	0.504
No.of oocytes retrieved	10.89 ± 2.99	11.17 ± 2.81	10.94 ± 2.93	11.09 ± 3.26	0.718	0.541
Fertilization method					1.342	0.719
IVF	820	234	328	141		
ICSI	145	43	49	27		

Notes: FSH represents follicle-stimulating hormone; LH represents luteinizing hormone; T represents testosterone; Gn represents gonadotropin; hCG represents human chorionic gonadotropin; IVF represents in vitro fertilization; ICSI represents intracytoplasmic sperm injection

^a^*P* < 0.05, compared with group A; ^b^*P* < 0.05, compared with group B

### Pregnancy outcomes and logistic regression analysis after embryo transfer in each group

As shown in Tables 3 and 4, there were no statistically significant differences in embryo quality, number of embryos transferred, or hCG positivity rates among the groups (*P* > 0.05). When comparing the LH/FSH < 2 group with the LH/FSH ≥ 2 group, there were no statistically significant differences in miscarriage rates or live birth rates between the two groups (*P* > 0.05). After chi-square testing, the miscarriage rates between the T ≤ 0.75 group and the T > 0.75 group were highly statistically significant [(11.38% vs. 18.50%) (*χ²* = 7.714, *P* = 0.006)], while the live birth rates were not significantly different [(61.28% vs. 57.79%) (*χ²* = 1.212, *P* = 0.271)]. Among the four groups of PCOS patients, the group with LH/FSH ≤ 2 and T > 0.75 had the lowest live birth rate and the highest miscarriage rate compared to the other three groups, with both differences being statistically significant (*P* < 0.05).

**Table 3:** Embryo quality and pregnancy outcome in each subgroup of patients

Item	Synchronized		Unsynchronized		F/χ2 value	*P* value
	Group A	Group B	Group C	Group D		
No. of transplant cycles	750	192	296	116		
D3 available embryo rate (%)	4115/5066 (82.20%)	1162/1385 (83.90%)	1666/2133 (78.11%)	742/949 (85.83%)	1.830	0.176
D3 good-quality embryo rate (%)	3198/5066 (63.88%)	903/1385 (65.20%)	1337/2133 (53.87%)	585/949 (61.64%)	0.436	0.509
D5 Blastocyst formation rate (%)	2804/5066 (56.01%)	808/1385 (58.34%)	1149/2133 (53.87%)	519/949 (54.69%)	9.566	0.069
No. of embryos transferred	1.57 ± 0.498	1.52 ± 0.501	1.52 ± 0.501	1.58 ± 0.496	1.332	0.263
Positive hCG rate (%)	559/750 (74.53%)	143/192 (74.48%)	219/296 (73.99%)	98/116 (84.48%	5.798	0.055
Abortion rate (%)	58/528 (10.98%)	30/134 (22.38%)^a^	25/201 (12.43%)^b^	12/93 (12.90%)^b^	9.41	0.02
Abortion rate (%)	88/662 (13.29%)		37/294 (12.58%)		0.09	0.764
Clinical pregnancy rate (%)	528/750 (70.4%)	134/192 (69.79%)	207/296 (69.93%)	93/116 (80.17%)	5.152	0.076
Live birth rate during transplantation cycle (%)	464/750 (61.87%)	99/192 (51.56%)^a^	177/296 (59.79%)^b^	79/116 (68.10%)^b^	9.870	0.02
Live birth rate during transplantation cycle (%)	563/942 (59.76%)		256/412 (62.13%)		0.673	0.412

Notes: FSH represents follicle-stimulating hormone; LH represents luteinizing hormone; T represents testosterone; hCG represents human chorionic gonadotropin

^a^*P* < 0.05, compared with group A; ^b^*P* < 0.05, compared with group B

The results of the multivariable logistic regression analysis showed that after adjusting for age, BMI, AFC, b E2, Gn dose, and Gn days, which were factors significantly influencing the outcome, the live birth rate in the LH/FSH < 2, T > 0.75 group (*OR* = 0.719, 95% *CI* = 0.520–0.994) had a significantly lower live birth rate than the LH/FSH < 2, T ≤ 0.75 group, and a significantly higher miscarriage rate (*OR* = 2.200, 95% *CI* = 1.325–3.653) (*P* = 0.002). The hCG positivity rate and clinical pregnancy rate were significantly higher in the LH/FSH ≥ 2, T > 0.75 group than in the LH/FSH < 2, T ≤ 0.75 group (*P* < 0.05), but there was no statistically significant difference in live birth rate and miscarriage rate between the two groups (*P* > 0.05).

**Table 4:** Logistic regression analysis of pregnancy outcomes in different subgroups

	Positive hCG rate	Clinical pregnancy rate	Abortion rate	Live birth rate
LH/FSH<2,T>0.75				
Before adjust OR (95% CI) *P*value After adjust OR (95% CI) *P*value	0.988 (0.997–1.434) 0.988 1.031 (0.714–1.488) 0.870	0.971 (0.688–1.373) 0.869 0.997 (0.704–1.413) 0.987	2.141 (1.301–3.522) 0.003 2.200 (1.325–3.653) 0.002	0.684 (0.497–0.941) 0.02 0.719 (0.520–0.994) 0.046
LH/FSH ≥ 2,T ≤ 0.75				
Before adjust OR (95% *CI*) *P* value After adjust OR (95% CI) *P* value	0.972 (0.715–1.321) 0.855 1.107 (0.792–1.548) 0.550	0.978 (0.729–1.312) 0.882 1.110 (0.807–1.527) 0.520	1.113 (0.676–1.834) 0.674 1.199 (0.712–2.019) 0.494	0.917 (0.695–1.207) 0.536 1.009 (0.752–1.355) 0.951
LH/FSH ≥ 2,T>0.75				
Before adjust OR (95% CI) *P* value After adjust OR (95% CI) *P* value	1.860 (1.096–3.157) 0.021 2.132 (1.210–3.756) 0.009	1.700 (1.049–2.755) 0.031 1.878 (1.127–3.132) 0.016	1.201 (0.618–2.334) 0.590 1.205 (0.597–2.432) 0.603	1.316 (0.867–1.998) 0.197 0.986 (0.952–1.021) 0.109
LH/FSH<2, T ≤ 0.75				
Ref.	Ref.	Ref.	Ref.	Ref.

Notes: FSH represents follicle-stimulating hormone; LH represents luteinizing hormone; T represents testosterone; hCG represents human chorionic gonadotropin

## Discussion

This study represents the first classification analysis of the characteristic indicators LH/FSH ratio and T levels in PCOS patients, providing certain clinical predictive value for IVF outcomes in this population. Our study further categorized the findings from previous studies and discovered that when the LH/FSH ratio is < 2 and T > 0.75, embryo quality remains unchanged, but the live birth rate decreases and the miscarriage rate increases. When the LH/FSH ratio ≥ 2 and T ≤ 0.75, there was no impact on outcomes. However, in the group with LH/FSH ≥ 2 and T > 0.75, while there was a positive effect on biochemical pregnancy and clinical pregnancy, there was no statistically significant difference in live birth and miscarriage rates. The study ultimately demonstrated that in PCOS patients with an unchanged LH/FSH ratio, elevated T levels may influence the final live birth outcome.

The elevated LH/FSH ratio in patients with PCOS is a typical characteristic of their neuroendocrine dysfunction, arising from an increased GnRH pulse frequency that leads to preferential pituitary synthesis of LH [20]. LH plays a clear dual role in follicular development: physiological levels of LH activity are necessary for optimal follicular growth, while excessively low levels suggest impaired hypothalamic-pituitary-ovarian axis function [21]. However, during controlled ovarian stimulation in assisted reproductive technology (ART), when LH exceeds a certain threshold, it may induce follicular atresia or premature granulosa cell luteinization, thereby inhibiting embryonic developmental potential [22]. Therefore, clinical interpretation requires a combined analysis of LH and FSH. Nevertheless, the predictive value of the LH/FSH ratio for ART outcomes exhibits significant heterogeneity in the existing literature. Some studies have shown that although an elevated basal LH/FSH ratio in PCOS patients is detrimental to natural ovulation, it is associated with higher clinical pregnancy and live birth rates, suggesting it may play a positive role in establishing pregnancy [16]. Conversely, other studies focus on its negative impacts: researchers found that while patients with LH/FSH ≥ 1 had higher rates of good-quality embryos, the cumulative clinical pregnancy rate was better in the group with LH/FSH ≤ 0.5 [23]. In patients undergoing in vitro maturation (IVM) treatment, those with LH/FSH > 1.5 had higher basal testosterone and antral follicle counts but lower pregnancy rates, potentially attributable to the adverse effects of LH on folliculogenesis and endometrial receptivity [24]. Simultaneously, a considerable number of studies have found no significant association: some researchers suggest that an elevated basal LH/ FSH ratio does not affect final pregnancy rates [13]; among PCOS patients undergoing GnRH antagonist protocols, the basal LH/FSH ratio had no significant impact on IVF/ICSI outcomes [13]; and for patients with LH/FSH ≥ 2, choosing either a GnRH agonist or a mild stimulation protocol did not alter clinical outcomes [25]. The results of the present study are consistent with this latter category, showing no significant differences in embryo quality or pregnancy outcomes among groups with different LH/FSH ratios. This heterogeneity suggests that the purported influence of the LH/FSH ratio may not represent a direct mechanistic effect, but rather an indirect reflection of different clinical phenotypes of PCOS. As a highly heterogeneous syndrome, PCOS presents with significant variations across different phenotypes in terms of androgen levels, ovarian reserve markers, and metabolic characteristics (including obesity, insulin resistance, and hyperinsulinemia) [26, 27]. These factors can independently influence oocyte quality and pregnancy outcomes [28, 29]. An elevated basal LH/FSH ratio might merely be a marker associated with a specific PCOS subtype, rather than a causal factor. The lack of significant association observed in this study may also be related to the specific phenotype distribution of the study population. In conclusion, while the LH/FSH ratio serves as an important window for understanding the neuroendocrine characteristics of PCOS, its predictive value may be limited in contemporary ART practice, as the widespread use of GnRH analogues potentially attenuates the impact of the endogenous endocrine state.

Androgens serve as precursors for estradiol (E2) biosynthesis and play an important role in normal follicular development [30]. However, the effects of androgens are concentration-dependent: studies have reported that abnormally high androgen levels can severely inhibit normal follicular development [31], subsequently affecting embryo quality and pregnancy outcomes. At the endometrial level, androgens play a decisive role in the decidualization of stromal cells, a process crucial for embryo implantation and placentation [32]. Clinical observations indicate that women with recurrent pregnancy loss have higher androgen levels than those with normal fertility, suggesting that elevated androgens may adversely affect endometrial function [33]. Animal experiments have further confirmed that high-dose androgens can interfere with endometrial development and the prostaglandin system, leading to early pregnancy loss [34]. Additionally, androgens participate in cervical remodeling and myometrial relaxation during normal labor, but excessively high levels may induce preterm birth [35].The predictive value of androgens for ART outcomes remains controversial in the existing literature. Some studies suggest that elevated androgen levels may lead to implantation-defect-related miscarriage by inhibiting the expression of estrogen-related genes in the endometrium [36]; however, other studies argue that while basal testosterone levels correlate with ovarian response, they cannot predict final IVF pregnancy outcomes [37–39]. Research specifically focusing on PCOS populations is relatively limited: recent studies have found that in PCOS patients undergoing GnRH antagonist protocols combined with freeze-all strategies, total testosterone and free testosterone have no significant impact on IVF outcomes [40]; conversely, other scholars have reported that in PCOS patients undergoing fresh embryo transfer, elevated T levels may increase the risk of early miscarriage and preterm birth, reducing live birth rates [41].The results of this study show that the trends in live birth rates (57.79% vs. 61.28%) and miscarriage rates (18.50% vs. 11.38%) between the high-T and non-high-T groups are generally consistent with the aforementioned literature [41]. Further analysis incorporating the LH/FSH ratio revealed that in the subgroup with LH/FSH < 2 and T > 0.75 ng/ml, the miscarriage rate was highest and the live birth rate lowest; however, this adverse effect was not observed in the subgroup with LH/FSH ≥ 2 and T > 0.75 ng/ml. Regarding this phenomenon, the coexistence of a higher LH/FSH ratio and elevated testosterone did not show additional adverse pregnancy outcomes, which may reflect the specific clinical or metabolic phenotypic characteristics of this subgroup (such as better ovarian reserve, lower insulin resistance, or a particular androgen profile), rather than a direct functional antagonism of the LH/FSH ratio on testosterone effects. At the same time, we also speculate that a higher LH/FSH ratio might partially “balance” the potential harm of high testosterone. However, this study did not observe significant differences in embryo quality or early assisted reproductive technology (ART) outcomes among groups with different LH/FSH ratios, suggesting that any hypothesis regarding a “balancing effect” lacks direct evidentiary support. On the other hand, in the ART setting, various physiological mechanisms are modulated by exogenous hormones or bypassed by ovarian stimulation protocols; therefore, hypotheses derived from physiology under natural conditions may not be directly applicable to the context of controlled ovarian stimulation. Currently, in clinical practice, it should be used as an auxiliary reference indicator, interpreted comprehensively in conjunction with the patient’s specific phenotype. Future research needs to incorporate refined phenotypic stratification and large-sample data to further elucidate the complex interactions among androgens, the LH/FSH ratio, and polycystic ovary syndrome (PCOS) subtypes, as well as their impact on ART outcomes.

The novelty of this study lies in its comprehensive assessment of basal LH, FSH, and T levels, offering a new analytical framework for research on assisted reproductive strategies in PCOS-related hyperandrogenism. This associative finding merely suggests a direction worthy of further exploration, and its value for clinical intervention requires confirmation through dedicated studies. However, it is important to note that the cutoff values for FSH, LH, and T levels may vary among different laboratories due to differences in testing methods and quality control standards. Each laboratory should establish its own cutoff values. A limitation of this study is that this study used chemiluminescence immunoassay to measure testosterone levels, which has limitations compared to mass spectrometry and may introduce potential measurement bias and T refers to serum total levels rather than free testosterone, the latter of which may be more reliable and/or specific in assessing hyperandrogenism. Additionally, we only considered the impact of baseline levels and did not assess whether sustained T levels post-embryo transfer or during pregnancy influenced embryo implantation and live birth. Furthermore, this study is a single-center retrospective study, so there may be bias in the baseline data, which has certain limitations. These findings still require further validation through studies with larger sample sizes or multicenter randomized prospective studies.

In summary, our findings suggest that the LH/FSH ratio alone may not be sufficient to reflect final pregnancy outcomes in patients with PCOS. However, combining it with testosterone levels could provide additional insight. Specifically, when both LH/FSH and testosterone levels are elevated, this combination does not appear to be associated with reduced live birth rates. However, in cases where the LH/FSH ratio is ≤ 2 and testosterone exceeds 0.75, there may be an association with lower live birth rates and higher miscarriage rates. Further studies are needed to validate these observations.

## Data Availability

All data presented in this study are available upon request upon contact with the corresponding author.
